# Sleep Quality Profiles in Youth with Eating Disorders: A Latent Profile Analysis

**DOI:** 10.3390/brainsci16050536

**Published:** 2026-05-19

**Authors:** Elvira Anna Carbone, Matteo Aloi, Renato de Filippis, Marianna Rania, Alessia Scordo, Claudia Procopio, Lavinia Rotella, Daria Quirino, Ettore D’Onofrio, Pasquale De Fazio, Cristina Segura-Garcia

**Affiliations:** 1 Department of Health Sciences, University Magna Graecia of Catanzaro, 88100 Catanzaro, Italy; elvira.carbone@unicz.it (E.A.C.);; 2 Department of Clinical and Experimental Medicine, University of Messina, 98125 Messina, Italy; matteo.aloi@unime.it; 3 Psychiatric Unit, University Hospital Renato Dulbecco, 88100 Catanzaro, Italy; 4 Department of Medical and Surgery Sciences, University Magna Graecia of Catanzaro, 88100 Catanzaro, Italy

**Keywords:** sleep quality, youth, eating disorders, latent profile, adolescent obesity, Pittsburgh Sleep Quality Index

## Abstract

**Highlights:**

**What are the main findings?**

- Sleep in youth with EDs shows distinct latent quality profiles.
- Four sleep profiles identified: less impaired, medication, poor, and insomnia-like.
- Profiles differ in eating psychopathology, distress, and emotion regulation.

**What are the implications of the main findings?**

- Profiles differ in emotion regulation, eating and general psychopathology.
- Night eating, diagnosis and adolescent obesity are associated with sleep profile membership.

**Abstract:**

Background/Objectives: Sleep disturbances are highly prevalent in young individuals with eating disorders (EDs) and are associated with increased psychopathology and poorer clinical outcomes. However, sleep alterations in ED populations are heterogeneous and may reflect distinct underlying clinical profiles. The study aimed to identify sleep quality profiles and examine their clinical correlates in youth with EDs. Methods: A total of 288 youth outpatients with EDs completed the Pittsburgh Sleep Quality Index (PSQI), along with measures of eating and general psychopathology. Latent Profile Analysis (LPA) was conducted using PSQI scores to identify distinct sleep profiles. Multinomial logistic regression models were performed to assess clinical variables of profile membership. Results: A four-profile solution was identified: (1) less impaired sleepers, (2) medication-using sleepers, (3) global poor sleepers, and (4) sleep-initiation-difficulty sleepers. Profiles differed significantly in ED severity, affective symptoms, emotion regulation difficulties, and sleep-related eating behaviors. Profiles characterized by greater sleep impairment exhibited higher levels of binge eating, night eating, and psychological distress. Multinomial logistic regression analyses indicated that night eating was the largest contributor to latent profile membership across all comparisons, significantly increasing the likelihood of belonging to more impaired sleep profiles. Conclusions: Sleep in individuals with EDs is characterized by distinct and clinically meaningful profiles rather than a uniform pattern of impairment. These findings support the clinical utility of person-centered approaches to better characterize sleep disturbances in ED populations.

## 1. Introduction

Sleep has been a central topic of scientific investigation for decades and plays a fundamental role in physical and psychological functioning, particularly during adolescence and young adulthood [1], a developmental period characterized by marked neurobiological, emotional, and behavioral vulnerability [2]. During this stage, sleep regulation undergoes substantial changes that often result in delayed circadian phase, reduced sleep duration, and increased susceptibility to sleep disturbances, which may interact with the onset or progression of psychiatric conditions [3,4,5,6].

Growing evidence suggests that individuals with eating disorders (EDs) frequently report impaired sleep quality, including difficulties in initiation, maintenance, and a globally non-restorative sleep compared to healthy controls [7], that, in turn, may worsen the clinical course of patients with EDs [7,8]. Eating habits and sleep are reciprocally related in multiple ways, not only through psychological and behavioral factors [9], but also through underlying neurobiological mechanisms (e.g., circadian rhythm regulation, REM sleep alterations, and new neuropeptides such as orexin) which may contribute to this bidirectional relationship [9].

Patients with EDs often engage in maladaptive eating behaviors (e.g., prolonged fasting, loss-of-control eating, and purging) that may negatively impact sleep quality. This suggests that key features of the eating-related psychopathology may be closely associated with alterations in sleep patterns [7] that may represent both maintaining factors and consequences of the disorder, potentially contributing to the persistence and severity of eating-related symptoms. Importantly, emerging evidence suggests that sleep disturbances in ED populations are not homogeneous. Rather than reflecting a uniform impairment, sleep alterations may vary considerably across individuals, potentially reflecting distinct underlying behavioral and psychopathological profiles [10]. Despite this, the heterogeneity of sleep disturbances across ED presentations remains poorly delineated. Given that EDs most commonly emerge during adolescence [11], there is a critical need to further investigate sleep disturbances within this population, adopting transdiagnostic approaches that move beyond categorical diagnoses to better capture underlying mechanisms.

To date, the literature has predominantly focused on anorexia nervosa (AN), with comparatively limited investigation of bulimia nervosa (BN) and binge eating disorder (BED). Traditional variable-centered approaches have provided valuable insights into average differences in sleep quality between clinical and non-clinical populations. However, these approaches may overlook meaningful inter-individual variability. In contrast, a person-centered approach, such as Latent Profile Analysis (LPA), allows for the identification of homogeneous subgroups of individuals based on shared patterns across continuous indicators, offering a more nuanced understanding of heterogeneity within clinical populations. LPA is a model-based clustering technique that identifies unobserved subgroups (latent profiles) by maximizing within-group similarity and between-group differences across continuous variables [12,13].

The Pittsburgh Sleep Quality Index (PSQI) is a validated multidimensional measure of subjective sleep quality that captures several domains of sleep functioning [14], thereby providing a suitable set of continuous indicators for person-centered approaches such as LPA. This approach assumes that individuals can be probabilistically assigned to qualitatively distinct profiles based on their response patterns, rather than being treated as members of a single homogeneous population [12].

To our knowledge, no studies have investigated sleep quality profiles derived from the PSQI in youth individuals with EDs in relation to general and eating-specific psychopathology using a person-centered framework. Addressing this gap may have important clinical implications, as identifying distinct sleep phenotypes may contribute to more precise characterization of ED heterogeneity and inform tailored intervention strategies.

The study aims to identify distinct sleep pattern profiles in youth with EDs using an LPA approach based on scores from the PSQI. The secondary objective is to examine whether the derived profiles differed in sociodemographic characteristics, ED diagnosis, and clinical features, including general and eating psychopathology. Finally, multinomial logistic regression analyses are conducted to examine the associations between clinical and psychopathological variables and latent profile membership. Given the exploratory nature of the investigation, no a priori hypotheses are formulated regarding either the number or the characteristics of the latent profiles.

## 2. Materials and Methods

### 2.1. Participants

The study included outpatients aged 14 to 30 years, diagnosed with EDs according to the Diagnostic and Statistical Manual of Mental Disorders, Fifth Edition (DSM-5) criteria [15], consecutively enrolled at the Outpatient Unit for Clinical Research and Treatment of Eating Disorders embedded within the Psychiatric Unit of the University Hospital “Renato Dulbecco” in Catanzaro (Italy), between April 2021 and February 2026, after providing informed consent (by parental or legal guardian for minors). Exclusion criteria included: inability to complete the self-report questionnaires, severe neurological disorders (e.g., traumatic brain injury, epilepsy); substance use disorder in the past six months; regular use of medications considered by the clinical team to substantially interfere with sleep architecture, circadian functioning, or eating behavior (e.g., sedative-hypnotics, antipsychotics, stimulant medications); age <14 or >30 years; refusal to provide consent. Occasional use of prescribed or over-the-counter sleep aids was allowed when use was sporadic and not part of an ongoing treatment regimen for a diagnosed sleep disorder.

### 2.2. Procedures

Assessments were conducted through clinical interviews administered by trained psychiatrists. Psychiatric diagnoses were confirmed using a structured clinical interview in accordance with the DSM-5 criteria [15]. An ad hoc form was completed to assess participants’ sociodemographic characteristics (i.e., age, sex, occupation, marital status, and education), anthropometric measures (i.e., weight, height, and body mass index [BMI]), and clinical characteristics. Participants completed the following set of questionnaires:

- Pittsburgh Sleep Quality Index (PSQI) [14] is a multidimensional self-report measure assessing different aspects of sleep functioning, including sleep quality, latency, duration, efficiency, medication, disturbances and daytime dysfunction. Higher scores indicate poorer sleep quality. A global PSQI score greater than 5 is generally considered indicative of poor sleep quality (ω 0.85);
- Eating Disorders Examination Questionnaire 6.0 (EDE-Q 6.0) [16] to evaluate eating psychopathology’s presence and severity across four subscales using 28 items: Eating Restraint, Eating Concern, Weight Concern, and Shape Concern, contributing to a global EDE score (ω 0.94);
- Binge Eating Scale (BES) [17] to investigate binge episodes severity, loss of control, emotional triggers using 16 items; scores of <17, 17–27 and >27 indicate that the risk of an individual having BED is unlikely, possible and probable, respectively (ω 0.87);
- Night Eating Questionnaire (NEQ) [18] is made up of 14 items that evaluate the night eating behavior (ω 0.77);
- Grazing Questionnaire (GQ) [19] consists of 8 items; higher scores reflect a greater degree of grazing behaviors and cognitions (ω 0.94);
- Beck Depression Inventory-II (BDI-II) [20] is a self-report instrument assessing the severity of depressive symptoms through 21 items. Higher scores indicate more severe symptoms. Scores fall into these standardized categories: <13: minimal depression; 14–19: mild depression; 20–28: moderate depression; >29: severe depression [21] (ω 0.82);
- State–Trait Anxiety Inventory (STAI S-T) is a self-report measure consisting of two 20-item subscales assessing state (STAI-S) and trait (STAI-T) anxiety [22]. Higher scores indicate greater anxiety (ω 0.88);
- Difficulties in Emotion Regulation Scale (DERS) [23] to evaluate multiple dimensions of emotion dysregulation (e.g., impulse control, awareness, strategies). The total score reflects overall problems in emotional regulation (ω 0.91);
- Morningness–Eveningness Questionnaire-reduced scale (MEQ-r) to assess preferred sleep–wake timing and peak periods of daily performance to categorize participants as morning, intermediate, or evening type [24] (ω 0.84);

This study adhered to the ethical principles outlined in the updated Declaration of Helsinki and obtained approval from the Ethical Committee of the “Regione Calabria, Sezione Area Centro” (identifier:162/22.04.2021).

### 2.3. Statistical Analysis

Statistical analyses were conducted using SPSS version 26.0 and R version 4.3.1, with the tidyLPA package [25], using the mclust backend. An LPA was performed to identify unobserved subgroups of individuals based on the seven PSQI component scores. Indicators were standardized prior to analysis. Models with one to six profiles were estimated using Model 1 parameterization in *tidyLPA*, which assumes equal variances across profiles and zero covariances among indicators (i.e., conditional independence). This specification was selected to ensure parsimony and interpretability. Model estimation was performed using the Expectation–Maximization (EM) algorithm. In the *mclust* framework, model initialization is based on model-based hierarchical clustering rather than random starting values. Convergence was evaluated based on successful model estimation and stability of the log-likelihood solution. Model selection was based on multiple statistical criteria, including the Akaike Information Criterion (AIC), Bayesian Information Criterion (BIC), sample size–adjusted BIC (saBIC), Consistent AIC (cAIC), Approximate Weight of Evidence (AWE), entropy, and the Bootstrap Likelihood Ratio Test (BLRT). Lower values of information criteria indicate better model fit, while higher entropy values reflect better classification accuracy. Classification quality was further evaluated using posterior probabilities, including the minimum posterior probability (prob_min). The optimal number of profiles was selected based on a combination of statistical fit indices, classification quality, profile size, and theoretical interpretability. After model selection, individuals were assigned to their most likely latent profile based on posterior probabilities.

Differences between profiles on self-report measures were examined using one-way analysis of variance (ANOVA) for continuous variables and chi-square (χ^2^) tests for categorical variables. When significant effects emerged, the Tukey post hoc test was performed. Effect sizes were calculated to complement statistical significance testing. Eta squared (η^2^) was used as a measure of effect size for ANOVA, whereas Cramer’s V was used for chi-square tests. Effect sizes were interpreted according to conventional thresholds. Internal consistency was assessed using McDonald’s ω, showing acceptable to excellent reliability across measures. Finally, exploratory multinomial logistic regression models were employed to examine the associations between latent profile membership and clinically relevant variables selected a priori based on previous literature on sleep disturbances and ED psychopathology (i.e., clinical characteristics, sleep quality, eating and general psychopathology) using standardized (z) scores. Multicollinearity among variables was assessed using Variance Inflation Factors (VIF) and tolerance values. All VIF values were below the conventional cutoff of 5, and all tolerance values were above 0.20, indicating no evidence of problematic multicollinearity among the variables included in the models. Statistical significance was set at *p* < 0.05.

## 3. Results

### 3.1. Latent Profile Analysis

Fit indices for models with one to six latent profiles are presented in Table 1. Across the estimated models, information criteria improved substantially from the one- to the four-profile solution, with more modest or worsening fit for additional profiles. The four-profile solution was selected as the optimal model based on a combination of statistical and substantive criteria. Specifically, this model showed the lowest values across multiple information criteria, including AIC (4911.61), BIC (5050.80), cAIC (5088.80), and sample size–adjusted BIC (4918.29), indicating superior model fit compared to both simpler and more complex solutions. The BLRT further supported the four-profile solution over the three-profile model (BLRT = 61.36, *p* = 0.0099), indicating that the addition of a fourth class significantly improved model fit. Although the BLRT remained significant for the five-profile solution, improvements in model fit were minimal and accompanied by a decline in classification quality and profile size. Classification quality for the four-profile solution was good, with entropy equal to 0.85 and minimum posterior probabilities above the recommended threshold (prob_min = 0.82), indicating adequate separation between latent classes [26]. All profiles were of sufficient size, with the smallest class representing approximately 15% of the sample, supporting the stability and interpretability of the solution. In contrast, models with a greater number of profiles showed progressively smaller class sizes and reduced classification precision. Taken together, these findings support the selection of the four-profile solution as the most parsimonious, statistically supported, and clinically interpretable representation of sleep patterns in the sample.

Profiles were interpreted based on standardized mean scores across PSQI components (sleep quality, latency, duration, efficiency, disturbances, medication use, and daytime dysfunction), with higher values indicating greater sleep impairment. It should be noted that the PSQI is a self-report measure and does not provide diagnostic classification of sleep disorders; accordingly, latent profiles are intended as descriptive patterns of sleep-related features rather than clinically defined sleep disorder categories.

**Figure 1:**
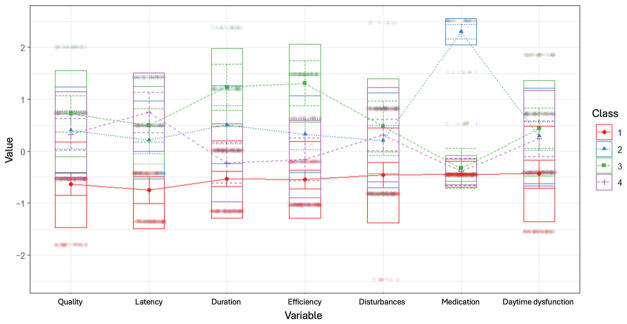
Graphic representation of mean-centered parameter estimates for PSQI profiles. Standardized group averages on PQSI subscales for a four-profile solution.

The four profiles were distributed as follows (N = 288): Profile 1 comprised 126 participants (43.8%), profile 2 included 43 (14.9%) patients, profile 3 included 45 participants (15.6%), and profile 4 included 74 patients (25.7%). Figure 1 displays standardized mean scores on the PSQI subscales across the four latent profiles.

#### 3.1.1. Profile 1—Less Impaired Sleepers

This was the largest group and was characterized by consistently below-average scores across all PSQI components, indicating minimal sleep-related difficulties. Individuals in this profile reported: better subjective sleep quality, shorter sleep latency, longer sleep duration and higher sleep efficiency, fewer sleep disturbances, low use of sleep medication, and reduced daytime dysfunction. This profile reflects a pattern of globally preserved and adaptive sleep functioning.

#### 3.1.2. Profile 2—Medication-Using Sleepers

This profile showed a highly distinctive pattern, characterized by self-reported elevated use of sleep aids, moderately elevated scores on sleep latency, duration, and efficiency, and slight increases in daytime dysfunction. This suggests a subgroup with clinically relevant sleep difficulties accompanied by recent or occasional use of sleep medications.

#### 3.1.3. Profile 3—Global Poor Sleepers

This group exhibited the highest levels of impairment across core sleep domains, including markedly elevated sleep duration problems, very poor sleep efficiency, increased sleep latency, elevated sleep disturbances and daytime dysfunction, and low use of sleep medication. This profile reflects a pattern of severe and pervasive sleep disturbances, notably in the absence of substantial pharmacological treatment.

#### 3.1.4. Profile 4—Sleep-Initiation-Difficulty Sleepers

This profile was characterized by elevated sleep latency (highest among profiles), moderately increased sleep disturbances and reduced sleep quality, near-average sleep duration and efficiency, low use of sleep medication, and moderate daytime dysfunction. This pattern may suggest moderate and domain-specific sleep difficulties, particularly related to sleep initiation.

Overall, the LPA revealed substantial heterogeneity in sleep patterns. Four clinically meaningful subgroups emerged: an adaptive sleep profile with minimal disturbances, a medication-using profile, a severely impaired and potentially under-treated profile, and a moderately impaired profile with selective difficulties. Notably, the most severely impaired group did not report elevated use of sleep aids, suggesting the presence of clinically significant sleep disturbances with low reported medication use.

### 3.2. Socio-Demographics Clinical Characteristics Across Profiles

Table 2 displays the sample demographic characteristics, also including the differences among the four latent profiles. A total of 288 participants were included in the study, with a mean age of 19.9 years (SD = 4.4). Most participants are female, single, and students attending middle or high school. Significant differences across profiles were found for civil status, with participants in Profile 3 more likely to be married or living with a partner compared to those in the other groups. No significant differences emerged for sex distribution, education or occupation that showed a similar distribution across the four profiles.

The distribution of the diagnostic categories across the four profiles is described in Table 3. A significant difference was observed among profiles (χ^2^ = 27.154, *p* = 0.001), with a small-to-moderate effect size (Cramer’s V = 0.177), indicating that diagnostic categories varied across sleep profiles. Participants in Profile 1 (less impaired sleepers) were more frequently diagnosed with AN restricting type (47.6%). Profile 2 (medication-using sleepers) showed a more heterogeneous distribution, with relatively higher proportions of BN (34.9%) and AN-binge-purging type (11.6%) compared with the other profiles. Profile 3 (global poor sleepers) was characterized by the highest proportion of BN (46.7%), whereas Profile 4 (sleep-initiation-difficulty sleepers) showed the highest prevalence of BED (40.5%). Overall, these findings suggest that different sleep profiles are associated with distinct patterns of ED diagnoses.

### 3.3. Psychopathological Assessment Across the Four Profiles

Table 4 depicts the psychopathological assessment of participants across the four latent sleep profiles. Significant differences emerged across profiles for all clinical variables. BMI showed a marginally non-significant difference (*p* = 0.059), with higher values in Profile 4 (sleep-initiation-difficulty sleepers). Self-report lifetime minimum and maximum body weight differed significantly across profiles (*p* = 0.019 and *p* = 0.018, respectively), with Profile 4 reporting higher values compared to Profiles 1 and 2. No significant differences emerged for obesity in early childhood (2–5 years) or late childhood obesity (6–12 years), whereas adolescent obesity (13–18 years) differed significantly across profiles (*p* = 0.031), with higher prevalence in Profiles 2 and 4. Eating disorder psychopathology (EDE-Q) showed significant group differences. In particular, Profile 1 (less impaired sleepers) consistently reported lower levels of EDE-Q subscales (restraint, eating concern, shape concern, and weight concern) and total scores compared to Profiles 3 and 4. Binge eating and night eating symptoms were also significantly different across groups. Scores on the BES and NEQ were higher in Profiles 3 and 4, with Profile 1 showing significantly lower levels compared to all other groups (for NEQ) and compared to Profiles 3 and 4 (for BES). Psychological distress variables showed robust differences. Depression (BDI-II), state and trait anxiety (STAI-S and STAI-T), and emotion dysregulation (DERS) were all significantly higher in Profiles 2, 3, and 4 compared to Profile 1. Finally, chronotype, assessed with the MEQ-r, differed significantly across profiles (*p* = 0.006), with a higher proportion of evening type in Profile 4.

Overall, these findings indicate that poorer sleep profiles, particularly Profiles 3 and 4, are associated with greater ED severity, binge and night eating behaviors, psychological distress, and evening chronotype.

### 3.4. Multinomial Logistic Regression

An exploratory multinomial logistic regression was performed to examine the association between latent profile membership and clinical variables, using Profile 1 (Less impaired sleepers) as the reference category (Table 5). The overall model indicated that night eating was the most consistent variable associated with profile membership.

#### 3.4.1. Profile 2 vs. Profile 1

Results showed that higher night eating significantly increased the likelihood of belonging to Profile 2 compared to Profile 1 (OR = 2.826, *p* = 0.023). In contrast, grazing was negatively associated with Profile 2 membership (OR = 0.365, *p* = 0.030), indicating that lower grazing scores were linked to a higher probability of belonging to this profile. All other variables were not significant.

#### 3.4.2. Profile 3 vs. Profile 1

Night eating was again a significant contributor to profile membership (OR = 3.066, *p* = 0.005), indicating higher levels of night eating in Profile 3 compared to Profile 1. Other variables, including depressive symptoms, BMI, anxiety and chronotype, were not significant.

#### 3.4.3. Profile 4 vs. Profile 1

Night eating remained a significant variable associated with profile membership (OR = 3.445, *p* < 0.001), indicating progressively higher levels of night eating in the most severe profile. Grazing showed a marginal negative effect (OR = 0.478, *p* = 0.056), while all other variables were not significantly associated with profile membership.

Overall, night eating emerged as the most constant contributor across all comparisons, significantly increasing the likelihood of belonging to profiles characterized by greater clinical severity, whereas grazing showed a weaker and less consistent (inverse) association.

## 4. Discussion

The present study identified four distinct sleep profiles in a clinical sample of adolescents and young adults with EDs using a person-centered LPA approach. The derived profiles reflected qualitatively different patterns of sleep functioning: (1) less impaired sleepers, (2) medication-using sleepers, (3) global poor sleepers, and (4) sleep-initiation-difficulty sleepers. Sleep profiles were derived from PSQI scores, a self-report measure without diagnostic capacity for sleep disorders; therefore, profiles reflect descriptive patterns of sleep-related characteristics rather than clinically defined sleep disorder categories. These findings support the presence of substantial heterogeneity in sleep disturbances within ED youth populations and extend prior research by demonstrating that sleep impairment is not uniform but instead clusters into clinically meaningful subgroups. Across profiles, significant differences emerged in ED diagnosis, psychopathological severity, emotion regulation, and sleep-related eating behaviors, highlighting the clinical relevance of sleep phenotypes in this population. Notably, night eating consistently emerged as the most robust contributor associated with latent profile membership, suggesting a central role of circadian-related eating dysregulation in the organization of sleep phenotypes among young individuals with EDs.

The identification of distinct sleep profiles provides empirical support for the notion that sleep disturbances in EDs represent a heterogeneous construct rather than a single-dimensional impairment. The less impaired sleepers’ profile exhibited more adaptive sleep functioning relative to other groups and lower levels of ED and general psychopathology, suggesting a more resilient subgroup despite the presence of a clinical ED diagnosis. In contrast, the “global poor sleepers” profile was characterized by severe and pervasive sleep disruption in the absence of substantial pharmacological treatment, accompanied by elevated levels of ED psychopathology and psychological distress. This pattern may suggest an association between greater sleep impairment and broader psychopathological burden, potentially contributing to illness severity and chronicity. The “medication-using sleepers” profile showed intermediate sleep impairment with sleep medication use, suggesting clinically relevant sleep difficulties potentially partially managed through recent or occasional use of sleep aids (e.g., hypnotics or sedative agents, which may partially improve sleep continuity without fully restoring physiological sleep architecture). Finally, the “Sleep-initiation-difficulty sleepers” profile was primarily characterized by marked sleep initiation difficulties, particularly prolonged sleep latency, alongside elevated emotional and eating-related psychopathology.

Interestingly, different sleep profiles were associated with distinct patterns of ED diagnoses. The AN-restrictive subtype was more frequently represented in the “less impaired sleepers” profile; however, this finding reflects a relative distribution within the latent profile classes rather than a clinical indication of preserved sleep in absolute terms. Indeed, individuals with AN-r were also present across all sleep profiles, highlighting the heterogeneity of sleep patterns within this diagnostic category. In contrast, binge-spectrum disorders (AN-bp and BN) were more evenly distributed across profiles, with higher proportions in the more impaired sleep classes, while BED showed a marked overrepresentation in the “insomnia-like sleepers” profile, suggesting a closer association with sleep initiation difficulties. The observed heterogeneity of individuals with AN across sleep profiles may be explained by differences in illness duration, stage of illness, and severity of malnutrition. Previous evidence has shown that AN is associated with poorer, non-restorative sleep compared to healthy controls [27], potentially driven by sleep fragmentation, malnutrition, low BMI, and associated systemic and neurobiological alterations affecting sleep regulation [28]. Sleep disturbances may further be influenced by reporting biases: patients with AN, often characterized by perfectionistic traits, may minimize sleep complaints despite engaging in wakefulness for goal-directed behaviors. In contrast, individuals with BN and BED may experience nocturnal binge eating and circadian disruption, behaviors that are often hidden due to shame and guilt [8]. From a transdiagnostic perspective, transitions across diagnostic categories may also reflect shifts in sleep architecture. Even subtle changes in symptom expression and clinical status may therefore influence subjective sleep experience, contributing to the perception of poorer sleep quality across ED populations compared to healthy controls.

Results of our study indicate that poorer sleep profiles are associated with greater ED severity, in particular, Profiles 3 and 4 showed higher levels of eating psychopathology (EDE-Q dimensions, BES, NEQ, GQ) compared to the more adaptive sleepers’ profile. This pattern may indicate a robust association between subjective sleep impairment and core eating-related cognitive and behavioral dysregulation, in particular, binge eating and night eating behaviors. Similarly, psychological distress, including depressive symptoms, anxiety, and emotional dysregulation, was significantly elevated in the more impaired sleep profiles, suggesting that sleep disturbances co-occur with broader affective and regulatory difficulties rather than representing an isolated symptom domain. Emotional dysregulation may further act as a maintaining mechanism, amplifying maladaptive coping strategies with negative emotions such as binge and night eating [29], and contributing to a bidirectional reinforcement between sleep disturbance and psychopathological distress. These findings further reinforce the close interplay between sleep quality and ED psychopathology. Particularly, obesity history did not differ in early or late childhood obesity, whereas adolescent obesity showed a significant association with sleep profiles, suggesting that sleep-metabolic interactions may be particularly relevant during developmental windows of vulnerability [30].

Of relevance, chronotype differences further supported a circadian component, with a higher proportion of evening types observed in the most impaired sleep profile. This pattern is consistent with evidence of eveningness in EDs [31] and broader delayed circadian phase shifts observed in psychiatric conditions [32], suggesting that circadian misalignment may contribute to both sleep disruption and maladaptive eating behaviors. In line with this framework, circadian dysregulation has been particularly implicated in bulimic-spectrum presentations, especially when binge eating occurs in the evening or at night, where eating behaviors may act as a chronobiological cue reinforcing wakefulness and delaying sleep onset [33], thereby potentially amplifying emotional and behavioral dysregulation. This is particularly relevant given that circadian system maturation is still ongoing across adolescence and young adulthood, potentially amplifying vulnerability to sleep–wake and eating dysregulation [34,35].

Another key finding of the present study is the robust and consistent association between night eating and latent sleep profiles. Night eating emerged as the most consistent variable linked to profile membership across all comparisons, increasing the likelihood of belonging to more impaired sleep profiles. This finding suggests that nocturnal eating behavior may represent a core behavioral marker linking sleep dysregulation and eating psychopathology. Grazing behaviors showed weak and predominantly inverse associations, suggesting that not all forms of disordered eating equally differentiate sleep-related profiles. This further supports the specificity of nighttime eating as a behavioral dimension closely linked to sleep–wake cycle alterations. From a mechanistic perspective, night eating has been associated with delayed circadian phase, impaired satiety signaling, and increased evening reward sensitivity, which may help explain disruptions in sleep–wake and feeding rhythms. In ED populations, these processes may be further exacerbated by factors such as emotional dysregulation and cognitive concern with food and body image, reinforcing maladaptive nocturnal behaviors [30]. These associations remained significant even after considering depressive symptoms, anxiety, BMI, and chronotype, suggesting that night eating may represent an important transdiagnostic dimension associated with sleep-related heterogeneity in ED populations. However, given the exploratory nature of the regression analyses and the conceptual overlap among several psychopathological measures, these findings should be interpreted cautiously and require replication in larger and more clinically stratified samples.

Overall, these results suggest that sleep profiles reflect a multidimensional clinical gradient spanning ED severity, emotional dysregulation, and circadian preference. Moreover, this pattern may support a transdiagnostic and dimensional interpretation of sleep disturbances, although sleep disruption should not be interpreted as a direct or exclusive marker of overall clinical severity.

### 4.1. Clinical Implications

The identification of distinct sleep phenotypes in ED patients may have potential clinical and research implications.

First, the observed heterogeneity in sleep profiles may suggest that routine clinical assessment of sleep in youth ED populations could benefit from considering more detailed evaluation of sleep–wake patterns and nocturnal eating behaviors, as well as circadian preference and sleep timing variability.

Second, the subgroup characterized by more severe sleep impairment and elevated night eating behaviors may represent an important target for future investigation. This highlights the need for further research on sleep disturbances in ED populations and more targeted assessment and intervention approaches addressing sleep disturbances.

Third, the association between sleep profiles and psychological distress underscores the potential utility of exploring integrated models of sleep, affective functioning, and eating psychopathology. Such models may inform future transdiagnostic research and the development of more comprehensive treatment approaches, once these phenotypes are replicated and validated.

### 4.2. Limitations

Several limitations should be considered. The cross-sectional design precludes causal inferences regarding the directionality of associations between sleep disturbances and psychopathology. The reliance on self-report measures may introduce reporting bias, and future studies should incorporate objective sleep measures such as actigraphy or polysomnography. A further limitation concerns the assessment of sleep medication use, which was based on self-reported PSQI items and did not allow for detailed information on drug type, dosage, or indication. In addition, the sample was clinically heterogeneous, spanning a wide age range (14–30 years), which includes both adolescents and young adults. This heterogeneity requires caution when interpreting the findings, as adolescence and young adulthood are characterized by partially distinct developmental trajectories in sleep timing, circadian preference, emotional regulation, and eating-related psychopathology. Developmental differences were not specifically examined and may have influenced sleep patterns and profile membership. Thus, future studies with larger samples should examine age-stratified or developmentally sensitive sleep profiles.

Furthermore, information on important clinical variables such as illness duration, treatment status, and comorbid psychiatric conditions was not consistently available and could not be included in the analyses. These factors may have contributed to the observed heterogeneity. Moreover, the sample was predominantly female, which may limit generalizability to male ED populations. Finally, although LPA provides a robust method for identifying latent subgroups, replication in independent samples is necessary to confirm the stability of the identified profiles, as well as their longitudinal stability over time.

## 5. Conclusions

In conclusion, the present study demonstrates that sleep patterns in EDs are not homogeneous but may cluster into distinct clinical phenotypes characterized by differential patterns of ED severity, psychological distress, and night eating behavior. Night eating emerged as a relevant feature distinguishing sleep profile, suggesting a potential transdiagnostic link between circadian dysregulation and eating pathology. These effects may be particularly pronounced in youth populations, in which developmental and circadian processes are still undergoing consolidation. These findings support the need to adopt a person-centered perspective in understanding sleep disturbances in EDs and may help inform future development of more individualized assessment and intervention approaches.

## Figures and Tables

**Table 1:** Absolute fit indices for measurement models.

Classes	AIC	AWE	BIC	cAIC	saBIC	Entropy	prob_min	prob_max	n_min	n_max	BLRT_val	BLRT_p
1	5742.141	5912.711	5793.429	5807.429	5749.034	1	1	1	1	1	NA	NA
2	5355.611	5625.051	5436.204	5458.204	5366.44	0.869358	0.934437	0.975413	0.298611	0.701389	402.5286	0.009901
3	4956.962	5384.019	5066.856	5096.856	4971.722	0.843262	0.905166	1	0.149306	0.503472	414.6518	0.009901
4	4911.606	5325.334	5050.796	5088.796	4918.293	0.853206	0.816496	1	0.149306	0.438028	61.36401	0.009901
5	4921.628	5466.959	5070.12	5116.12	4924.247	0.828228	0.734927	1	0.097222	0.371528	25.98012	0.029703
6	4950.365	5564.357	5098.163	5152.163	4926.921	0.802851	0.71349	1	0.0625	0.34375	17.26058	0.138614

AIC: Akaike Information Criteria; AWE: Approximate Weight of Evidence; BIC: Bayesian Information Criteria; cAIC: Consistent AIC; saBIC: sample size–adjusted BIC; BLRT: Bootstrap Likelihood Ratio Test.

**Table 2:** Characteristics of the total sample and by latent profiles.

			Profile 1	Profile 2	Profile 3	Profile 4			
		Total Sample	Less Impaired Sleepers	Medication- Using Sleepers	Global Poor Sleepers	Sleep-Initiation-Difficulty Sleepers	Statistic		Effect Size
Variable		M (SD)	M (SD)	M (SD)	M (SD)	M (SD)	F	*p*	η^2^
Age		19.9 (4.4)	19.2 (4.0)	19.8 (4.5)	20.1 (4.3)	20.9 (5.0)	2.299	0.078	-
		N (%)	N (%)	N (%)	N (%)	N (%)	*χ^2^*	*p*	*Cramer’s V*
Sex	Female	268 (93.0)	119 (94.4)	39 (90.7)	43 (95.6)	67 (93.0)	1.906	0.592	-
	Male	20 (7.0)	7 (5.6)	4 (9.3)	2 (4.4)	7 (7.0)			
Civil status	Single	267 (92.7)	122 (97.6)	38 (95.0)	40 (88.9)	67 (94.4)	18.550	**0.029**	0.148
	Married	8 (2.8)	2 (1.6)	0 (0.0)	2 (4.4)	4 (5.6)			
	Co-habitant	5 (1.7)	1 (0.8)	1 (2.5)	3 (6.7)	0 (0.0)			
	Divorced	1 (0.3)	0 (0.0)	1 (2.5)	0 (0.0)	0 (0.0)			
	NA	7 (2.4)	-	-	-	-			
Education	Elementary school	2 (0.7)	0 (0.0)	0 (0.0)	0 (0.0)	2 (2.7)	8.583	0.477	-
	Middle school I	140 (48.6)	58 (46.0)	25 (58.1)	23 (51.1)	34 (46.6)			
	High school II	122 (42.4)	58 (46.0)	16 (37.2)	18 (40.0)	30 (41.1)			
	University Degree	23 (8.0)	10 (7.9)	2 (4.7)	4 (8.9)	7 (9.6)			
	NA	1 (0.3)	-	-	-	-			
Occupation	Unemployed	31 (10.8)	16 (13.2)	1 (2.6)	3 (6.8)	11 (15.9)	19.889	0.069	-
	Employed	18 (6.2)	7 (5.8)	1 (2.6)	6 (13.6)	4 (5.8)			
	Unpaid activity	3 (1.0)	0 (0.0)	0 (0.0)	2 (4.5)	1 (1.4)			
	Self-employed	7 (2.4)	4 (3.3)	0 (0.0)	2 (4.5)	1 (1.4)			
	Student	213 (73.0)	94 (77.7)	36 (94.7)	31 (70.4)	52 (75.4)			
	NA	16 (5.56)	-	-	-	-			

NA: not available. Significant results are in bold.

**Table 3:** The frequencies of eating disorder diagnosis across the four profiles.

	Profile 1	Profile 2	Profile 3	Profile 4			
	Less Impaired Sleepers	Medication- Using Sleepers	Global Poor Sleepers	Sleep-Initiation-Difficulty Sleepers	Statistic		Effect Size
Diagnosis	N (%)	N (%)	N (%)	N (%)	χ^2^	*p*	Cramer’s V
AN-r	60 (47.6)	16 (37.2)	16 (35.6)	19 (25.7)	27.154	**0.001**	0.177
AN-bp	5 (4.0)	5 (11.6)	1 (2.2)	2 (2.7)			
BN	31 (24.6)	15 (34.9)	21 (46.7)	23 (31.1)			
BED	30 (23.8)	7 (16.3)	7 (15.6)	30 (40.5)			
Total	126 (100.0)	43 (100.0)	45 (100.0)	74 (100.0)			

AN-r: Anorexia nervosa-restricting type; AN-bp: Anorexia nervosa-binge purging type; BN: bulimia nervosa; BED: binge eating disorder. Significant result is in bold.

**Table 4:** Clinical characteristics of the total sample and by latent profiles.

	Profile 1	Profile 2	Profile 3	Profile 4				
	Less Impaired Sleepers	Medication- Using Sleepers	Global Poor Sleepers	Sleep-Initiation-Difficulty Sleepers	Statistic		Effect Size	Post Hoc
Variable	M (SD)	M (SD)	M (SD)	M (SD)	F	*p*	η^2^	
BMI	22.6 (9.3)	23.1 (7.4)	23.0 (9.1)	26.4 (9.9)	2.513	0.059	-	-
Minimum body weight	49.6 (14.6)	47.9 (15.0)	50.8 (16.3)	56.4 (16.0)	3.365	**0.019**	0.040	1,2 < 4
Maximum body weight	68.2 (22.7)	72.3 (25.3)	72.1 (27.1)	80.8 (26.9)	3.435	**0.018**	0.041	1 < 4
EDE-Q Restraint	2.9 (1.9)	3.4 (2.2)	3.8 (1.8)	3.5 (1.8)	3.380	**0.019**	0.036	1 < 3
EDE-Q Eating concern	2.9 (1.6)	3.3 (1.4)	3.8 (1.4)	3.7 (1.3)	6.496	**<0.001**	0.067	1 < 3,4
EDE-Q Shape concern	4.2 (1.7)	4.8 (1.4)	5.0 (1.0)	5.0 (1.3)	5.666	**<0.001**	0.059	1 < 3,4
EDE-Q Weight concern	3.6 (1.8)	4.2 (1.6)	4.5 (1.4)	5.6 (1.4)	6.238	**<0.001**	0.065	1 < 3,4
EDE-Q Tot	3.4 (1.5)	4.0 (1.5)	4.3 (1.1)	4.2 (1.3)	7.013	**<0.001**	0.072	1 < 3,4
BES Tot	17.6 (12.3)	21.5 (10.2)	25.1 (11.1)	24.4 (10.1)	7.637	**<0.001**	0.079	1 < 3,4
NEQ Tot	12.2 (6.4)	16.3 (7.4)	19.2 (7.3)	18.6 (6.5)	19.956	**<0.001**	0.177	1 < 2,3,4
GQ Tot	9.5 (7.0)	9.4 (6.7)	12.1 (6.6)	12.1 (6.1)	3.623	**0.014**	0.038	1 < 4
BDI-II Tot	22.1 (12.5)	32.9 (12.9)	32.8 (12.7)	33.3 (11.5)	13.777	**<0.001**	0.164	1 < 2,3,4
STAI-S Tot	49.9 (13.3)	60.3 (10.6)	59.4 (11.4)	58.0 (11.4)	13.029	**<0.001**	0.124	1 < 2,3,4
STAI-T Tot	52.6 (12.5)	59.7 (11.0)	62.1 (9.2)	61.4 (10.0)	13.685	**<0.001**	0.130	1 < 2,3,4
DERS Tot	101.7 (30.9)	118.8 (25.7)	120.3 (28.5)	124.5 (24.4)	11.897	**<0.001**	0.117	1 < 2,3,4
MEQ-r Tot	14.9 (3.8)	14.3 (4.5)	13.7 (3.6)	12.6 (4.0)	4.268	**0.006**	0.057	1 < 4
	N (%)	N (%)	N (%)	N (%)	χ^2^	p	Cramer’s V	
MEQ-r chronotype								
Intermediate	61 (69.3)	17 (56.7)	24 (68.6)	37 (60.7)	14.053	**0.029**	0.181	
Morning	16 (18.2)	7 (23.3)	3 (8.6)	4 (6.6)				
Evening	11 (12.5)	6 (20)	8 (22.9)	20 (32.8)				
Early childhood obesity	22 (20.0)	7 (21.2)	7 (17.9)	17 (26.1)	1.275	0.735	-	
Late childhood obesity	41 (37.3)	12 (36.4)	13 (33.3)	33 (50.8)	4.382	0.223	-	
Adolescent obesity	47 (42.7)	18 (54.5)	14 (35.9)	40 (61.5)	8.848	**0.031**	0.189	

BDI-II: Beck Depression Inventory-II; BES: Binge Eating Scale; BMI: Body Mass Index; DERS: Difficulties in Emotion Regulation Scale; EDE-Q: Eating Disorder Examination Questionnaire; GQ: Grazing Questionnaire; MEQ-r: Morningness–Eveningness Questionnaire; NEQ: Night Eating Questionnaire; STAI-S/T: State–Trait Anxiety Inventory. Significant results are in bold.

**Table 5:** Multinomial logistic regression.

Parameter Estimates (Coefficients)					Exp(B) 95% Confidence Intervals			
Response	Name	Estimate	SE	Exp(B)	Lower	Upper	z	*p*
2–1	(Intercept)	−0.94	0.32	0.39	0.21	0.74	−2.90	0.004
	BMI	−0.03	0.36	0.97	0.47	1.97	−0.096	0.924
	STAI-T	0.44	0.56	1.56	0.52	4.71	0.80	0.426
	EDE-Q tot	−0.04	0.39	0.96	0.44	2.07	−0.11	0.913
	BDI-II	0.91	0.51	2.49	0.90	6.87	1.77	0.076
	BES	0.15	0.52	1.16	0.42	3.22	0.28	0.776
	NEQ	1.04	0.46	2.83	1.15	6.98	2.27	**0.023**
	GQ	−1.01	0.46	0.36	0.15	0.91	−2.17	**0.030**
	DERS	−0.14	0.51	0.87	0.32	2.38	−0.28	0.781
	MEQ-r tot	0.20	0.32	1.22	0.65	2.27	0.62	0.534
3–1	(Intercept)	−0.82	0.31	0.44	0.24	0.81	−2.64	0.008
	BMI	−0.53	0.37	0.59	0.29	1.22	−1.44	0.150
	STAI-T	0.70	0.56	2.01	0.67	6.03	1.25	0.211
	EDE-Q tot	0.02	0.42	1.02	0.44	2.37	0.06	0.953
	BDI-II	0.15	0.52	1.16	0.41	3.27	0.28	0.776
	BES	0.62	0.48	1.87	0.73	4.81	1.31	0.190
	NEQ	1.12	0.40	3.07	1.40	6.74	2.82	**0.005**
	GQ	−0.26	0.43	0.77	0.33	1.81	−0.61	0.542
	DERS	−0.24	0.48	0.79	0.31	2.04	−0.49	0.621
	MEQ-r tot	−0.04	0.29	0.97	0.54	1.73	−0.12	0.902
4–1	(Intercept)	−0.34	0.27	0.71	0.411	1.22	−1.26	0.209
	BMI	0.11	0.29	1.11	0.630	1.96	0.37	0.711
	STAI-T	0.69	0.52	2.00	0.715	5.59	1.33	0.183
	EDE-Q tot	−0.27	0.37	0.76	0.367	1.59	−0.73	0.465
	BDI-II	0.42	0.46	1.53	0.611	3.82	0.91	0.360
	BES	0.43	0.43	1.54	0.664	3.58	1.02	0.309
	NEQ	1.24	0.37	3.45	1.643	7.23	3.31	**<0.001**
	GQ	−0.74	0.39	0.48	0.223	1.03	−1.91	0.056
	DERS	0.39	0.43	1.48	0.638	3.45	0.92	0.356
	MEQ-r tot	−0.06	0.27	0.94	0.552	1.59	−0.24	0.810

BDI-II: Beck Depression Inventory-II; BES: Binge Eating Scale; BMI: Body Mass Index; DERS: Difficulties in Emotion Regulation Scale; EDE-Q: Eating Disorder Examination Questionnaire; GQ: Grazing Questionnaire; MEQ-r: Morningness–Eveningness Questionnaire; NEQ: Night Eating Questionnaire; STAI-T: Trait Anxiety Inventory. Significant results are in bold.

## Data Availability

The data presented in this study are available on request from the corresponding author due to privacy reasons.

## Abbreviations

The following abbreviations are used in this manuscript:

AN-r	Anorexia nervosa-restricting type
AN-bp	Anorexia nervosa-binge purging type
BDI-II	Beck Depression Inventory-II
BED	Binge eating disorder
BES	Binge eating scale
BMI	Body mass index
BN	Bulimia nervosa
DERS	Difficulties in Emotion Regulation Scale
DSM-5	Diagnostic and Statistical Manual of Mental Disorders, Fifth Edition
EDs	Eating disorders
EDE-Q 6.0	Eating Disorder Examination Questionnaire 6.0
GQ	Grazing Questionnaire
LPA	Latent profile analysis
MEQ-r	Morningness–Eveningness Questionnaire-reduced scale
NEQ	Night Eating Questionnaire
PSQI	Pittsburgh Sleep Quality Index
STAI-S/T	State–Trait Anxiety Inventory
VIF	Variance Inflation Factors
